# Looking Back at 2017 with ASTMH Past President Patricia Walker: Political Challenges Remind Her that “Optimism is a Moral Imperative”

**DOI:** 10.4269/ajtmh.17-interview

**Published:** 2018-01

Patricia F. Walker, MD, DTM&H, FASTMH, Professor of Medicine at the University of Minnesota, Medical Director of the HealthPartners Travel and Medicine Center, and a leading authority on refugee health, recently completed her term as ASTMH President for 2016–2017. She sat down with science writer Matthew Davis to discuss what was often a tumultuous time for tropical medicine and global health.

## Looking back at your year as president, what are some of the challenges and achievements that stand out for you, both within the Society itself and in the broader world of global health?

I think the most significant thing that impacted global health and tropical medicine over the last year was the election of Donald Trump as President. That made us, as a Society, realize the importance of calm, steady advocacy for tropical medicine and global health funding. Things we thought never would be slated for elimination, like the Fogarty International Center, or threatened with drastic cuts, like the NIH and the CDC, showed us that we have a responsibility to be speaking to the public and to our elected leaders about the importance of what we do.

We spent a lot of time this year advocating on behalf of global health and tropical medicine. I’m really proud that ASTMH was seen as an expert voice in Washington and internationally. And as a scientific society, we took a nonpartisan approach, talking about the evidence for the things that we think are very important, like the role of open borders for encouraging scientific exchange and scientific diplomacy and why that is essential to our global health security agenda.

Also, as someone who has devoted a career to refugee health and migration medicine, I am really proud that the Society stepped forward after the first travel ban was initiated by the Trump administration to speak about best practices in immigration policies. We were right at the forefront with an immediate response that required a fast turnaround and quick action from our Executive Committee.

## Did this kind of work on the front lines of intense national political debates take many in the Society out of their comfort zones?

Most of us feel comfortable within our scientific disciplines. We are not trained to be speaking and writing as advocates. On the other hand, as I said in my Presidential Address, I believe we are frontline witnesses to health inequities around the world. We often know that the issue is not a shortage of good science or good clinical options, it’s that we don’t have the political will to solve big problems in global health. I agree with what Paul Farmer said in his keynote address at the Annual Meeting, that many of the problems we experience in global health persist due to insufficient investments and attention, not because we lack scientific insights.

## Over the last few years the Ebola outbreak and then the Zika outbreak generated considerable interest in infectious diseases and global health challenges. Is there a danger that, with these threats no longer front and center, we are drifting back to business-as-usual until the next emergency comes along?

I think from the perspective of the Society, we have taken the lessons we learned from the Ebola and Zika outbreaks and are using them to continue improving how we deal with these kinds of challenges. I think the work by (ASTMH Scientific Program Chair) Dan Bausch is a good example. We have learned from these experiences and must continue to change and adapt. What concerns me is how the political problems of the last year have caused us to divert attention from important issues like preparing for the next disease outbreak.

## Given these challenges—the fact that major initiatives have been threatened and many believe science itself is being devalued—how does one stay enthusiastic about addressing problems in global health?

I really believe that at times like these, optimism is a moral imperative. If you feel you can have any impact on your own future or the future of your patients or your scientific research, you have to be an optimist. You also have to take the long view that solving problems like social justice, equal access to health care, and ridding the world of tropical diseases is not going to happen in your lifetime. But there can be significant incremental change. When we started a clinical practice at HealthPartners Center for International Health in Minnesota 37 years ago, we had to deal with providers who did not know the differential diagnosis for tropical diseases seen in immigrants and were sometimes racist. But when I look back now, I can see that we have come a really long way. It requires balance to continue to have a heightened sense of outrage, yet maintain that long view; it helps me keep my patience today in what are very difficult times.

## What do you see as some of the key challenges and opportunities the Society will encounter in the coming year?

The Society will need to maintain a strong focus on advocating for global health R&D and for evidence-based decision making on issues that affect global health, like immigration policy. I also think the Society needs to keep working to mentor the next generation of scientists and to make sure our international members feel supported. We now have two elected international members on the Council. That’s not enough, but we are making progress. We are planning a regional meeting in Mali and we want to do more with our digital education work so that our international members, who may not be able to afford to attend trainings, can access educational materials via webinars and other online options.

I know that (new ASTMH President) Regina Rabinovich will keep the conversation going around gender issues within the Society. I’m very proud of the Ludlow Medal (the Clara Southmayd Ludlow Medal, the first Society-level medal to be named after a female icon in tropical medicine). We have done a very good job with gender balance in the Annual Meeting symposia, with invited speakers and the Council. We need more attention to Society-level awards and medals, where recipients mostly have been men.

Another exciting thing is to have a Clinical Tropical and Travel Medicine Education Program Chair position in the Society, which is something new. Our membership includes the world’s experts in the field of tropical medicine and we need to be leveraging that knowledge to train the next generation. John Sanders and his committee are exploring with the American Board of Internal Medicine the possibility of establishing a subspecialty in clinical tropical medicine.

Overall, I think the Society is in a really good position today. We are poised to continue to grow both in terms of size and influence, and we will maintain that visionary goal of working toward a world free of tropical infectious diseases.

**Figure:**
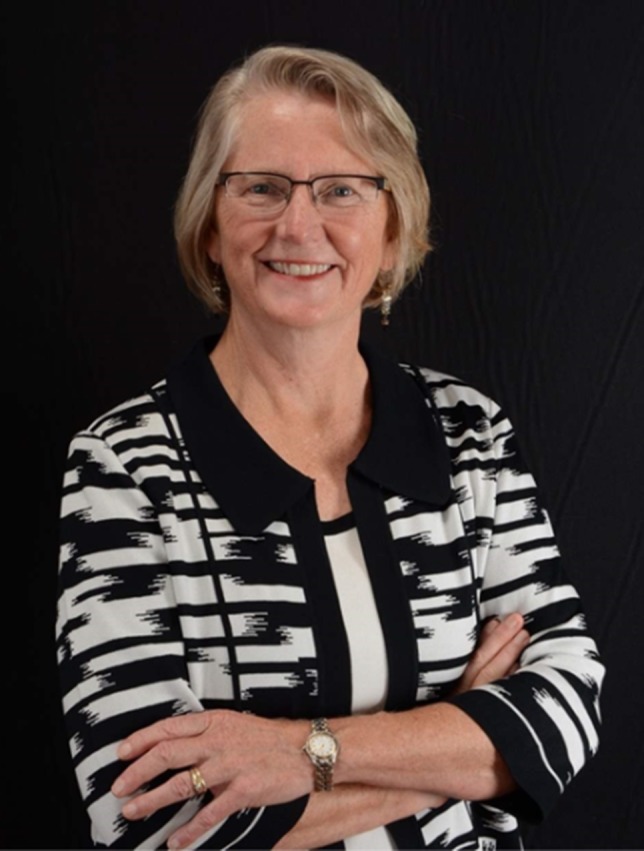
**2017 President Patricia F. Walker, MD, DTM&H, FASTMH**

